# The FYVE Domain of Smad Anchor for Receptor Activation (SARA) Is Required to Prevent Skin Carcinogenesis, but Not in Mouse Development

**DOI:** 10.1371/journal.pone.0105299

**Published:** 2014-08-29

**Authors:** Huang-Ming Chang, Yu-Ying Lin, Pei-Chun Tsai, Chung-Tiang Liang, Yu-Ting Yan

**Affiliations:** 1 Program in Molecular Medicine, National Yang-Ming University and Academia Sinica, Taipei, Taiwan, ROC; 2 Institute of Biomedical Sciences, Academia Sinica, Taipei, Taiwan, ROC; 3 Institute of Biochemistry and Molecular Biology, National Yang-Ming University, Taipei, Taiwan, ROC; 4 National Laboratory Animal Center, National Applied Research Laboratories, Taipei, Taiwan, ROC; Cedars-Sinai Medical Center, United States of America

## Abstract

Smad Anchor for Receptor Activation (SARA) has been reported as a critical role in TGF-β signal transduction by recruiting non-activated Smad2/3 to the TGF-β receptor and ensuring appropriate subcellular localization of the activated receptor-bound complex. However, controversies still exist in previous reports. In this study, we describe the expression of two SARA isoforms, SARA_1_ and SARA_2_, in mice and report the generation and characterization of SARA mutant mice with FYVE domain deletion. SARA mutant mice developed normally and showed no gross abnormalities. Further examination showed that the TGF-β signaling pathway was indeed altered in SARA mutant mice, with the downregulation of Smad2 protein expression. The decreasing expression of Smad2 was caused by enhancing Smurf2-mediated proteasome degradation pathway. However, the internalization of TGF-β receptors into the early endosome was not affected in SARA mutant mouse embryonic fibroblasts (MEFs). Moreover, the downregulation of Smad2 in SARA mutant MEFs was not sufficient to disrupt the diverse cellular biological functions of TGF-β signaling, including growth inhibition, apoptosis, senescence, and the epithelial-to-mesenchymal transition. Our results indicate that SARA is not involved in the activation process of TGF-β signal transduction. Using a two-stage skin chemical carcinogenesis assay, we found that the loss of SARA promoted skin tumor formation and malignant progression. Our data suggest a protective role of SARA in skin carcinogenesis.

## Introduction

The TGF-β signaling pathway is involved in many cellular processes, including cell growth, differentiation, migration, immunosuppression, and the epithelial-to-mesenchymal transition (EMT) [1]–[3] in developing embryos and adult organisms. It is also associated with a variety of pathological conditions, such as fibrosis and cancer [4], [5]. Signal transduction begins with the binding of TGF-β ligand to a specific receptor complex that consists of type II and type I serine/threonine kinase receptors (TβRII and TβRI). In the complex, phosphorylation of the type I receptor by the constitutively active type II receptor leads to receptor activation. The phosphorylated type I receptor then binds and phosphorylates its downstream signal-mediators, R-Smad proteins (Smad2 and Smad3). Once phosphorylated, R-Smads dissociate from the receptor complex and associate with the co-Smad, Smad4. The R-Smad/Smad4 complexes translocate to the nucleus where they bind to distinct DNA binding proteins and regulate the transcription of specific target genes [6]–[8]. It has been widely accepted that the scaffold protein Smad Anchor for Receptor Activation (SARA) facilitates the activation process of the TGF-β signaling [9].

SARA, Smad Anchor for Receptor Activation, also is the zinc finger FYVE domain -containing protein 9 (*ZFYVE9*). The N-terminal FYVE domain of SARA binds with high specificity to the phosphoinositol-3′-phosphate (PtdIns3P) and localizes SARA to the phospholipid-containing membrane. PtdIns3P is highly enriched in the early endosome; thus, the FYVE domain can mediate the localization of SARA to this endocytic compartment. The central region of SARA contains a Smad-binding domain (SBD) that interacts with unphosphorylated Smad2 and Smad3. In addition to binding Smads, SARA also binds the TGF-β type I and II receptor complex via a large C-terminal domain. Therefore, SARA was suggested to work as a scaffold protein to bring Smad2/3 to TGF-β receptors and facilitate Smad2/3 activation [9]–[11]. Furthermore, both ectopic expression of the dominant-negative FYVE domain of SARA and knockdown of SARA expression by siRNA can inhibit TGF-β signaling [12], [13]. Thus, SARA was hypothesized to be critical for the initiation and maintenance of TGF-β signaling.

In the canonical TGF-β signaling pathway, SARA transduces TGF-β signaling by controlling the phosphorylation and localization of Smad2/3. A recent study has shown that long-term treatment with TGF-β results in diminishing the expression of SARA in cells and leads to the downregulation of Smad2 protein to abrogate Smad2-dependent transcriptional responses. Notably, loss of SARA does not affect Smad3 protein expression or TGF-β/Smad3 signal transduction. Because loss of Smad2 expression makes cells more permissive for the EMT progression, these results suggest a functional role of SARA in the modulation of EMT processing [13].

Although the biochemical role of human SARA in TGF-β signaling has been intensively studied in cell culture systems, several contradictory findings have been reported recently [14]–[16]. Therefore, the precise biochemical and biological roles of SARA *in vivo* need to be further explored. Here, we report the tissue specific expression pattern of SARA and generate the SARA FYVE domain deficient (SARA-dFYVE) mice to verify the necessity and significance of this protein *in vivo*. Our results indicate that mouse SARA plays a key role in preventing Smad2 degradation via Smurf2-mediated proteasomal degradation pathway, rather than participates in the regulation of TGF-β signaling transduction process. The downregulation of Smad2 in SARA mutant mice contributes to increased skin tumor formation and malignant conversion, but does not affect mouse embryonic development.

## Materials and Methods

### Whole-mount *in situ* hybridization

Mouse embryos from embryonic day (E) 7.0 to E10.5 were analyzed for SARA expression by whole-mount *in situ* hybridization with digoxigenin-labeled RNA probes. Briefly, the antisense RNA probes for the mouse SARA N-terminal (nt 1 to 500) and C-terminal (nt 3695 to 4194) domains were synthesized by *in vitro* transcription. The fragments of SARA cDNA used for RNA probe synthesis were amplified from a mouse brain cDNA preparation. The primer pairs (SARA-E1-f: atggagaattacttccaagc and SARA-E2-r: atgagggattgactattgta; SARA-E14-f: cccaggaacagatccacatc and SARA-E17-r: ctatgcgatgttttccagaa) were used for SARA N-terminal and C-terminal cDNA amplification, respectively. *In situ* hybridization was performed as described previously [17].

### Generation of SARA FYVE domain deficient mice

**Figure 1:**
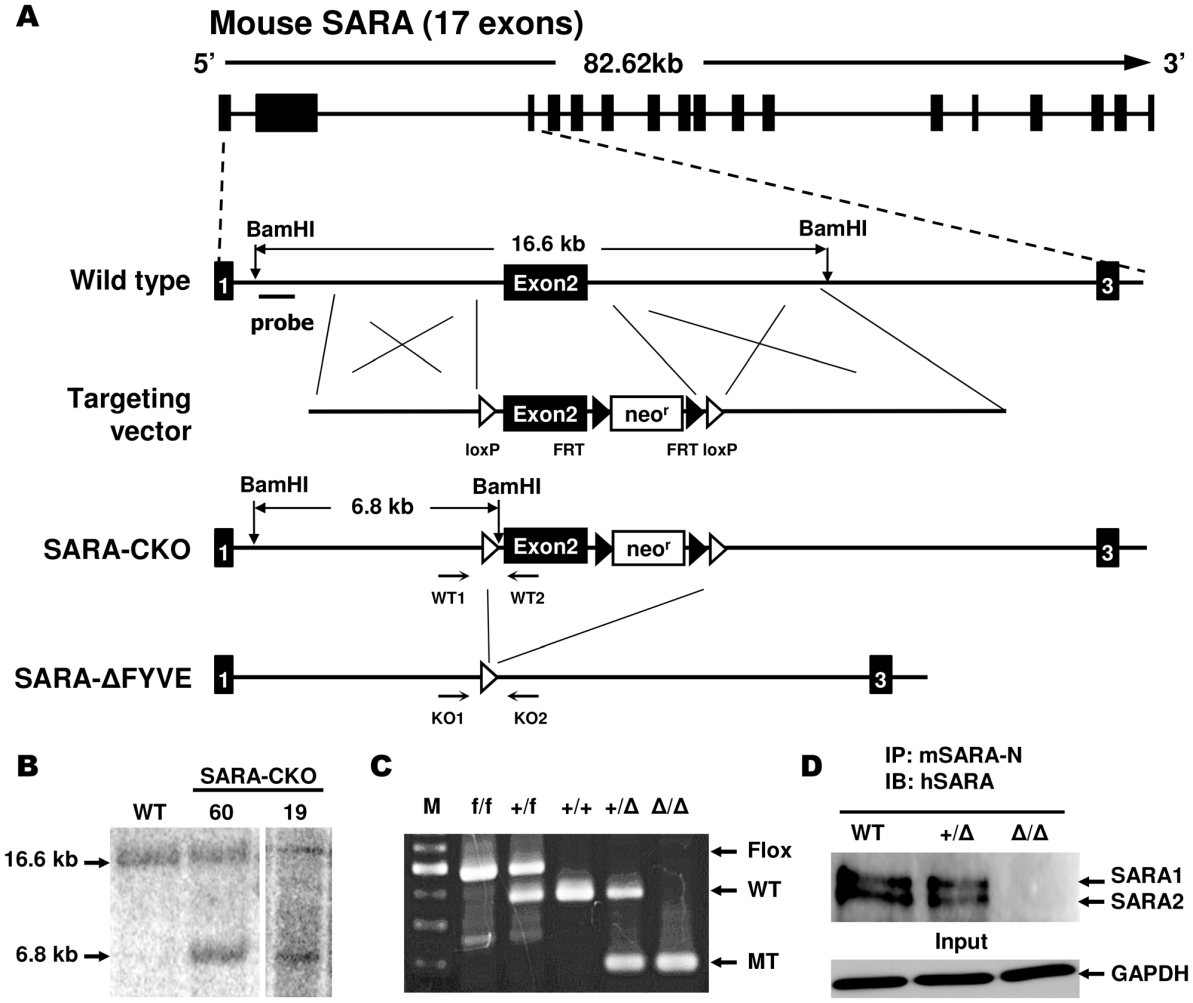
Generation of SARA FYVE domain deficient mice. (A) Schematic diagram of the SARA genomic locus, targeting construct, and genomic locus of the resulting SARA-dFYVE mutant mice after Cre-mediated deletion. The numbered black boxes are SARA exons. The open and black arrowheads indicate loxP and FRT sites, respectively. Neor is the neomycin resistance cassette. The flanking probe used in Southern blotting and the expected fragment sizes after BamHI digestion of wild type (16.6 kb) and mutant (6.8 kb) genomic DNA are indicated. The locations of the PCR primers used to screen genotypes are shown (arrows). (B) ES cell clones with correctly disrupted alleles were confirmed by Southern blot analysis with the 5′ external probe indicated in panel A. Two independent SARA-CKO ES clones (#19 and #60) were identified. (C) Genotyping of SARA-dFYVE mutant mice by PCR using the primers (WT1 and WT2) or (KO1 and KO2) are shown in panel A. (D) Total protein lysates (0.5 mg) from MEFs were immunoprecipitated (IP) with anti-mouse SARA-N antibody and then blotted (IB) with anti-human SARA antibody. The expression of SARA1 and SARA2 proteins was completely abolished in SARAΔ/Δ mice. Western blotting with the GAPDH antibody served as the input control.

Mouse SARA contains 17 exons; the FYVE domain of SARA is located within exon 2. A targeting vector for SARA conditional knockout (SARA-CKO) mice was constructed by inserting loxP sequences into intron 1 and intron 2. A FRT-flanked neomycin resistance (neo^r^) cassette was also inserted downstream of exon 2. The FYVE domain of SARA was removed following recombination by Cre protein. Linearized SARA CKO-targeting vector was delivered into 129S6/SvEvTac-derived TC1 embryonic stem (ES) cells by electroporation. Gene targeting of SARA in ES cells resulted in an extra BamHI site at the SARA recombinant allele (Figure 1A). For Southern blot analysis, genomic DNA extracted from ES cells was digested with BamHI and hybridized with the probe indicated in Figure 1A. This probe identifies a 16.6-kb fragment and a 6.8-kb fragment in the wild type (WT) and SARA-CKO alleles, respectively. SARA-CKO ES cells from these individual clones were injected into C57BL/6 blastocysts. The resulting SARA-CKO chimeric males were subsequently crossed with C57BL/6 females. To generate SARA-dFYVE mutant mice, the mice carrying the SARA-CKO allele were crossed with protamine-Cre transgenic mice [18] to delete the entire exon 2. Genotyping was performed by PCR with primer pairs for the WT and SARA-CKO allele (WT1: tactgtatagatttagcaaa and WT2: ggcagtggttgtgcatgtc) and for the SARA-dFYVE mutant allele (KO1: tttcacttcaggctcccaag and KO2: catgccctgctgtaagttgg). All animals were maintained on a mixed 129S6/SvEvTac and C57BL/6 background.

### Preparation of mouse embryonic fibroblast (MEF) cells and cell culture

Primary WT and SARA-dFYVE mutant MEFs were derived from E13.5 embryos and maintained in Dulbecco's modified Eagle's Medium (DMEM), supplemented with 10% fetal bovine serum (FBS) and 1% penicillin-streptomycin stock solution (10,000 U/mL penicillin; 10,000 µg/mL streptomycin) at 37°C in an atmosphere containing 5% CO_2_. For the TGF-β-induction assay, MEFs were serum-starved for 24 hours in medium containing 0.1% FBS. After serum starvation, the cells were treated with 4 or 10 ng/mL of TGF-β1 (R&D) for the indicated times. For proteasome inhibition assay, MEFs were treated with proteasome inhibitor MG132 (20 µM) or DMSO as a negative control for 8 hours.

### RT-PCR and Quantitative real-time PCR

Total RNA was extracted from adult mouse brain and kidneys using TRIzol reagent (Invitrogen). Reverse transcription reactions were performed at 42°C using the BioScript cDNA synthesis kit (Bioline). The fragments of SARA_1_ and SARA_2_ cDNA were amplified with the following primer pairs: (SARA-E1-f: atggagaattacttccaagc and SARA-E2-r: atgagggattgactattgta) or (SARA-E2-f: tttcaaaggaacttgcatga and SARA-E17-r: ctatgcgatgttttccagaa), respectively. The fragments of SARA_3/4_ cDNA were amplified with the following primer pair: (SARA-E1-f: atggagaattacttccaagc and SARA-E5-r: tgacacagccagagttcctg). GAPDH served as a loading control with the primer pair (GAPDH-f: cttcaccaccatggagaagg and GAPDH-r: ggcatggactgtggtcatgag). Quantitative real-time PCR (Q-PCR) was performed using SYBR Green PCR master mix (Roche) and the following primer pairs: (Smad2-f: cgaggttttgaagccgttta and Smad2-r: tgggtttacgacatgcttga) and (Tubulin-f: ccattggcaaggagatcattg and Tubulin-r: atggcctcattgtctaccatg). The relative mRNA expression levels were calculated according to the ΔΔ*Ct* method and normalized to Tubulin.

### Generation of mouse SARA-N and -C antibodies

The cDNA encoding the N-terminus (a.a. 1 to 100) and C-terminus (a.a. 1098 to 1397) of mouse SARA was cloned into pGEX-4T1 and pPAL7 vectors, respectively. The recombinant proteins were expressed in *Escherichia coli* strain BL21. Purified SARA-N and -C inclusion bodies were sent to LTK BioLaboratories and used as immunogens for the production of polyclonal antibodies in guinea pigs.

### Immunoprecipitation and Western blot analysis

For immunoprecipitation experiments, adult mouse tissue specimens or MEF cells were homogenized with RIPA buffer (50 mM Tris, pH 7.4, 150 mM NaCl, 1% Nonidet P-40, 0.25% Na-deoxycholate, 0.1% SDS, 1 mM PMSF, 1 mM NaF, 1 mM Na_3_VO_4_, and protease inhibitor cocktail (Roche)). Tissue (1 mg) or cell lysates (0.5 mg) were incubated with various antibodies overnight at 4°C and immunoprecipitated with Protein G beads (Millipore). Bound complexes were resolved on 8% gels and analyzed by Western blotting. The following commercial primary antibodies were used for immunoprecipitation and/or Western blots: Smad2 (Abcam), phospho-Smad2 (Cell Signaling), Smad3 (Cell Signaling), phospho-Smad3 (Cell Signaling), TGF-β RI (Santa Cruz), TGF-β RII (Santa Cruz), human-SARA (Santa Cruz), Smurf2 (Cell Signaling), and GAPDH (Millipore). Antibodies to mouse-SARA-N and mouse-SARA-C were generated in our laboratory. Incubation with anti-mouse IgG HRP, anti-rabbit IgG HRP, anti-guinea pig IgG HRP (Jackson Lab), and TrueBlot anti-rabbit HRP antibodies (eBioscience) followed by ECL was used for detection.

### Immunocytochemistry and immunohistochemistry

For immunocytochemistry, serum-starved MEFs were grown on glass slides, treated with TGF-β1, fixed with cold 4% paraformaldehyde (PFA) for 15 min, and then incubated in NH_4_Cl/PBS (50 mM) for 10 minutes. Cells were permeabilized with 0.25% Triton X-100, blocked in 3% BSA for 30 minutes at room temperature, and incubated with primary antibodies at 4°C overnight. Cells were immunolabeled with the following antibodies: Smad2 (Santa Cruz), Smad3 (Cell Signaling), α-SMA (Sigma), TGF-β RI (Santa Cruz), TGF-β RII (Santa Cruz), and EEA1 (BD Biosciences). FITC- or rhodamine-conjugated secondary antibodies were used, and nuclei were counterstained with Hoechst 33342 (Invitrogen). Mounted cells were analyzed by confocal laser scanning microscopy using a Zeiss LSM Meta 510. For immunohistochemistry, skin samples were fixed in 4% PFA, embedded in paraffin, and sectioned. Sections were heated in DAKO Target Retrieval Solution (pH 6.0) for antigen retrieval, blocked in 5% BSA for 1 hour at room temperature, and incubated with Smad2 (Santa Cruz) primary antibody at 4°C overnight. Samples were incubated with HRP-conjugated secondary antibody and exposed to DAB for colorimetric detection. Nuclei were counterstained with hematoxylin. Mounted samples were analyzed by digital fluorescent microscopy.

### Cell proliferation and senescence-associated β-gal assays

Cell proliferation was measured by the MTT [3,4-(5-dimethylthiazol-2-yl)-5-(3-carboxymethoxyphenyl)-2-(4-sulfophenyl)-2H-tetrazolium salt] assay [19]. Briefly, approximately 1000 cells of early-passage MEFs were plated in triplicate in 96-well plates and cultured in MEF medium with or without 4 ng/mL TGF-β1. Cell proliferation was determined at 48 hours intervals by adding 50 µl MTT reagent (2 mg/mL). After incubation of cells at 37°C for 2 hours, DMSO was added, and the absorbance at 492 nm was measured. For senescence assays, WT or SARA^Δ/Δ^ MEFs were either left untreated or treated with 4 ng/mL TGF-β1 for 6 days. Cells were then fixed and stained with β-galactosidase [20].

### Skin chemical carcinogenesis protocol

Two-step skin carcinogenesis was induced by DMBA and TPA. The back-skins of 8-week-old mice were shaved and treated with DMBA (20 µg in 50 µl acetone) for tumor initiation. One week later, TPA (5 µg in 50 µl acetone) was applied topically to skin twice a week for 20 weeks for tumor promotion. The number of tumors per mouse was recorded weekly [21].

### Pathological evaluation of skin tumors

Skin tumors were fixed in 4% PFA, embedded in paraffin, sectioned, and stained with H&E. The tumor types were classified according to the following criteria: ulceration may be present; cellular squamous differentiation is variable; size and staining of nuclei is variable; loss of intercellular bridges is present; atypical, bizarre mitotic figures are present; mitotic figures are numerous; invasion of the dermis and striated muscle by nests or cords of squamous cells is present; basal lamina is penetrated by invasive growth [22].

### Ethics statement

All animal experiments were approved by the Institutional Animal Care and Use Committee of Academia Sinica (Protocol ID: 11-11-243). All tumor burdened animals were euthanized when they had reached ethical endpoints. The determination of ethical endpoints was when the mice found unexpectedly to be moribund, cachectic, or unable to obtain food or water.

## Results

### Analysis of SARA expression

**Figure 2:**
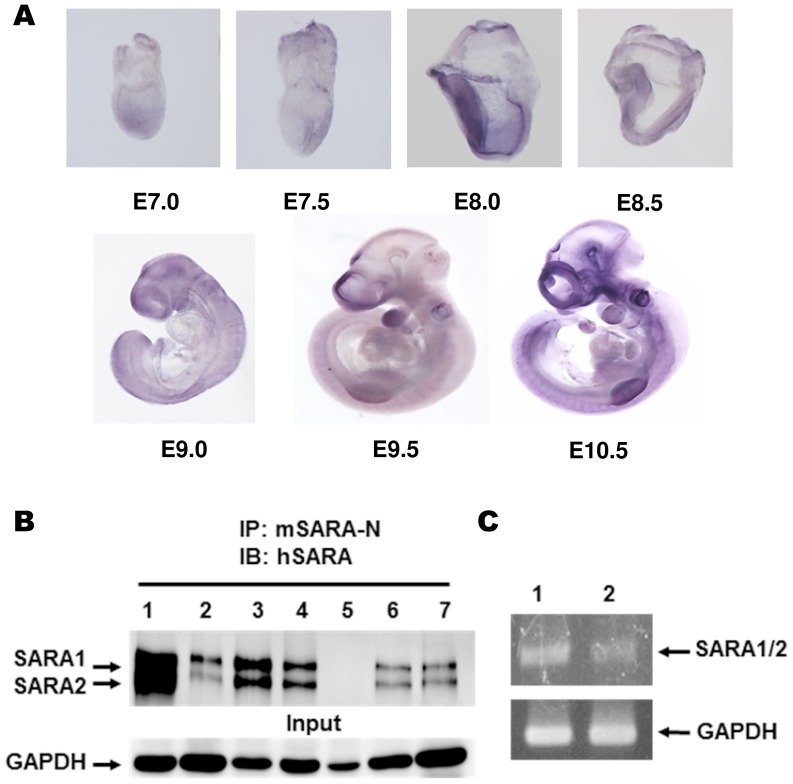
SARA expression patterns in embryonic and adult mice. (**A**) SARA transcripts were detected in the mouse embryos at embryonic day (E) 7.5 to E10.5 by whole-mount *in situ* hybridization. (**B**) Western blotting was performed to evaluate the expression of SARA in each adult mouse tissue. Total protein lysates (1 mg) were immunoprecipitated (IP) with anti-mouse SARA-N antibody and then blotted with anti-human SARA antibody. Adult mouse tissues are as follows: brain (lane 1), heart (lane 2), lung (lane 3), liver (lane 4), kidney (lane 5), spleen (lane 6), and skin (lane 7). Two isoform of SARA proteins (SARA_1_ and SARA_2_) were detected in each tissue except the kidney. Expression of GAPDH was used as the input control. (**C**) RT-PCR analysis of adult brain (lane 1) and kidney (lane 2) mRNAs was performed using the primer pair (SARA-E1-f and SARA-E2-r) for SARA_1/2_ transcripts. SARA_1_ and SARA_2_ transcripts were detected in adult mouse kidneys. GAPDH served as the input control.

Although the biochemical role of SARA has been intensively studied, the biological role of SARA *in vivo* still remains unknown. Thus, we used a mouse model to explore the biological function of SARA. Even though SARA has been identified as a ubiquitously expressed gene [9], the embryonic and tissue-specific expression patterns of SARA during embryonic and adult stages are not well established. We first performed whole-mount *in situ* hybridization analysis to determine the SARA mRNA expression pattern during early development. The results of whole-mount *in situ* hybridization using either N-terminal (Figure 2A) or C-terminal (data not shown) SARA RNA probes indicate that SARA mRNA transcripts are expressed in many cell types at early embryonic stages.

**Figure 3:**
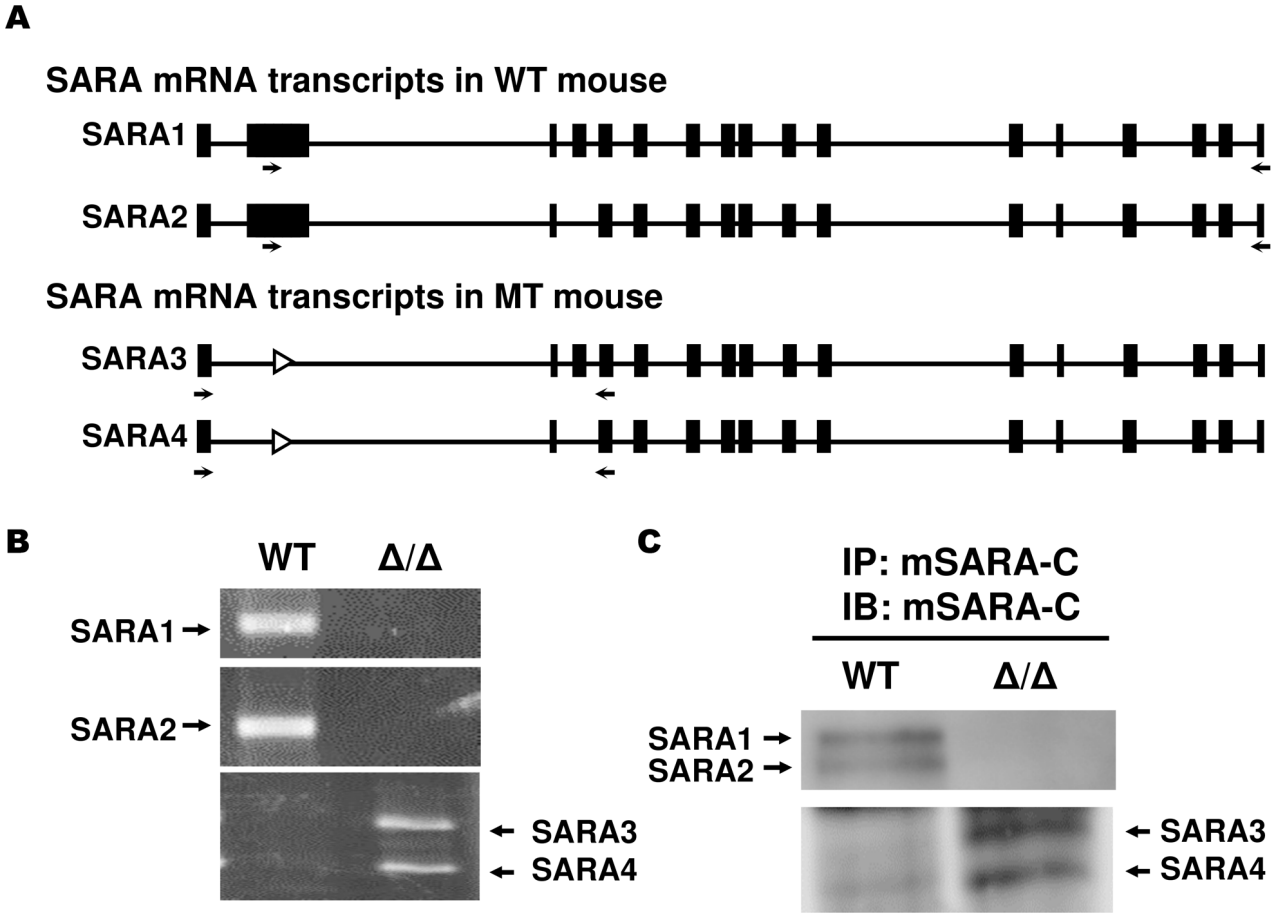
Expression of truncated SARA proteins in SARA mutant mice. (A) Schematic diagram of the mouse SARA mRNA transcripts in WT and SARA-dFYVE mutant mice. The black boxes are SARA exons and the open arrowheads indicate loxP sites. The locations of the RT-PCR primers used to detect SARA mRNA transcripts are shown (arrows). (B) RT-PCR analysis of mouse brain total RNA, performed using the primer pair (SARA-E2-f and SARA-E17-r) for SARA1 and SARA2 transcripts and the primer pair (SARA-E1-f and SARA-E5-r) for SARA3 and SARA4 transcripts, is shown in panel A. (C) To confirm that truncated SARA proteins (SARA3 and SARA4) were expressed in SARA-dFYVE mutant mice, Total protein lysates (1 mg) from adult skin was immunoprecipitated (IP) and blotted (IB) with anti-mouse SARA-C antibody.

In the Ensemble database (http://www.ensembl.org/index.html), we found three predicted mouse SARA proteins (Protein ID: ENSMUSP00000102268; ENSMUSP00000102269; ENSMUSP00000039852), denoted SARA_1_, SARA_2_, and SARA_3_, respectively. The SARA_1_ transcript, the longest one, contains 17 exons and produces a polypeptide with 1397 1397 amino acids (a.a.). The SARA_2_ transcript lacks exon 4, which contains a Smad-binding domain (SBD), and it encodes a protein with 1338 a.a. The SARA_3_ transcript lacks exon 2, which contains the FYVE domain, and it encodes a protein with 706 a.a. The mouse SARA_1_ and SARA_2_ transcripts are closely related to two human SARA isoforms (Protein ID: ENSP00000360647; ENSP00000349737), respectively; humans do not have a transcript similar to the mouse SARA_3_ isoform. In adults, SARA proteins are expressed at a low level in mouse tissues. However, the SARA proteins could be detected by Western blot analysis following enrichment by immunoprecipitation. We detected two isoforms of mouse SARA protein (SARA_1_ and SARA_2_) with molecular weights around 200 kDa in most mouse tissues except the kidney (Figure 2B). However, RT-PCR results indicated that both SARA_1_ and SARA_2_ transcripts were present in the mouse kidney (Figure 2C). Surprisingly, we could not detect any mouse SARA_3_ mRNA transcript or protein in WT mouse tissues and cells (Figure 3B and 3C). This predicted transcript variant, which does not encode the important FYVE domain of SARA, may not be present in mice. Our data indicate that mouse SARA only has two isoforms (SARA_1_ and SARA_2_), which are expressed ubiquitously in mice during embryonic stages and through adulthood.

### Generation of SARA FYVE domain deficient mice

TGF-β signaling controls embryonic development, and the loss of TGF-β components often leads to embryonic or perinatal lethality [23]–[26]. To avoid possible embryonic lethality caused by SARA mutant, we used gene targeting and the Cre/loxP system to generate SARA conditional knockout (CKO) mice. The full-length gene encoding SARA contains 17 exons and spans approximately 82.62 kb on chromosome 4 (Figure 1A). Briefly, we generated mice harboring loxP sites flanking exon 2 of SARA. Exon 2 encodes the N-terminal half of the SARA protein and includes the FYVE domain, which is the key functional domain of SARA. Furthermore, the deletion of exon 2 by Cre protein results in a frameshift mutation in the SARA gene. ES clones that had undergone homologous recombination were identified by Southern blot analysis using an external hybridization probe. Several targeted ES cell clones were identified and used to generate two SARA-CKO mouse lines (Figure 1B). To establish a conventional SARA mutant mouse line, SARA-CKO mice were crossed with protamine-Cre transgenic mice, which excise the floxed region at an early stage of spermatogenesis [18]. Homozygous mutant mice (SARA^Δ/Δ^) were generated by intercrossing two heterozygous (SARA^+/Δ^) mice. Mice were genotyped using genomic DNA extracted from mouse tails with specific primer pairs (Figure 1C). Interestingly, normal Mendelian ratios of WT, SARA^+/Δ^, and SARA^Δ/Δ^ were observed from the intercross of SARA^+/Δ^ mice, indicating that SARA_1_ and SARA_2_ are not necessary for early mouse development and postnatal survival (Table S1). The detailed histopathological analysis also revealed that adult SARA^+/Δ^ and SARA^Δ/Δ^ mice developed normally and showed no significant abnormalities compared with WT mice (data not shown). In addition, the litter sizes of offspring from the intercross of SARA^Δ/Δ^ mice were normal, indicating SARA is not required for fertility. Western blot analysis revealed that the expression of SARA_1_ and SARA_2_ in SARA^+/Δ^ MEFs was reduced to approximately one-half of that in WT cells, but was absent in SARA^Δ/Δ^ MEFs (Figure 1D). Thus, the important N-terminal FYVE domain of SARA is dispensable for mouse development and is not needed for viability or fertility.

To verify that SARA mutant mice expressed the truncated form of mutant SARA or not, RT-PCR analysis was performed using total mRNA from adult mouse brain with the specific primer pairs indicated in Figure 3A. SARA_1_ and SARA_2_ mRNAs were expressed in WT mice, but SARA_3_ was not. In SARA^Δ/Δ^ mice, after deletion of the exon 2 sequence, SARA_1_- and SARA_2_-modified mRNAs were still present and were converted into SARA_3_ and SARA_4_ truncated transcripts (Figure 3A–B). The SARA_4_ transcript lacks exon 2 and exon 4, which encode the N-terminal FYVE and Smad binding domain (SBD), respectively. To confirm that SARA_3_ and SARA_4_ transcripts were translated into N-terminal truncated proteins, SARA proteins were examined in skin tissues of WT and SARA^Δ/Δ^ adult mice. Western blot analysis was performed using a specific mouse SARA-C antibody that recognizes SARA_1_, _2_, _3_, and _4_ simultaneously (Figure 3C). The results of Western blot analysis showed that SARA_1_ and SARA_2_ were expressed in WT but not SARA^Δ/Δ^ mice. However, N-terminal truncated SARA_3_ and SARA_4_ proteins could still be detected in SARA^Δ/Δ^ mice. The complete amino acid sequences for mouse SARA isoforms are given in Text S1.

### TGF-β/Smad2 signaling is downregulated in SARA mutant mice

**Figure 4:**
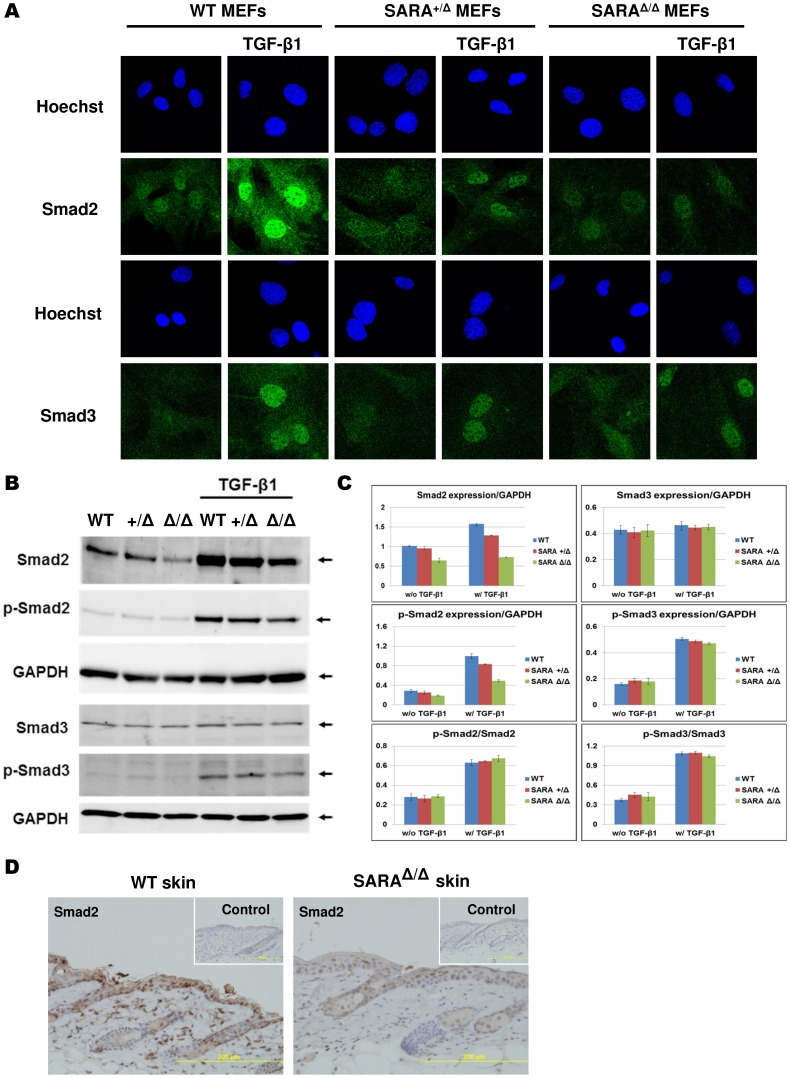
TGF-β/Smad2 signaling is downregulated in SARA mutant mice. (**A**) Serum-starved MEFs were treated with or without 4 ng/mL TGF-β1 for 30 min. The cellular locations of Smad2 and Smad3 were detected by immunofluorescence staining using Smad2 and Smad3 antibodies. TGF-β-induced nuclear translocation of Smad2 but not Smad3 was decreased in SARA mutant MEFs. (**B**) After cells were treated with or without 4 ng/mL TGF-β1 for 1 hour, MEF lysates were collected and analyzed by Western blot. Total Smad protein and phosphorylated Smad protein were detected by specific antibodies as indicated. (**C**) Quantification of Western blot results in panel B showed that mutation of SARA protein did not alter the ability of Smad2 protein phosphorylation. Although Smad2 protein levels were reduced, Smad3 expression was not altered. (**D**) Analysis of Smad2 expression in mouse skin by immunohistochemistry with Smad2 antibody. The controls were incubated with only the secondary antibody, as shown in the insert sections. Smad2 protein was decreased in SARA^Δ/Δ^ skin compared with WT.

To examine the integrity of the TGF-β signaling pathway in SARA mutant mice, TGF-β-induced Smad2/3 activation was determined by the extent of its nuclear localization in MEFs using Smad2 and Smad3 antibodies. In the absence of TGF-β stimulation, endogenous Smad2 and Smad3 in MEFs primarily localized to the cytoplasm, although a small amount of protein was also detected in the nucleus. Following TGF-β activation, Smad2 rapidly accumulated in the nucleus of WT MEFs. In contrast, Smad2 nuclear translocation was decreased in SARA^+/Δ^ and SARA^Δ/Δ^ MEFs (Figure 4A). Fluorescence immunocytochemical analysis results showed that the nuclear localization of Smad2 was abrogated in SARA mutant MEFs. Notably, following TGF-β activation, the nuclear localization of Smad3 in SARA mutant MEFs was not changed as in WT MEFs (Figure 4A).

In addition, the results of Western blot analysis were consistent with the findings of the fluorescence immunocytochemical assay. Levels of TGF-β-induced phosphorylated Smad2 (p-Smad2) were decreased in SARA mutant MEFs. Moreover, the total amount of Smad2 protein was also reduced in SARA mutant MEFs (Figure 4B). By contrast, downregulation of p-Smad3 or Smad3 total protein did not occur (Figure 4B). Quantitative analysis of Western blot results further revealed that the ratio between p-Smad2 and Smad2 total protein was not changed (Figure 4C), which indicates the loss of the FYVE domain of SARA did not affect the ability of TGF-β to induce Smad2 phosphorylation. Significant downregulation of Smad2 expression was also observed in the skin tissue of SARA^Δ/Δ^ mice by immunohistochemical staining with Smad2 antibody (Figure 4D). Therefore, the downregulation of Smad2 protein is not limited to SARA mutant MEFs.

### SARA does not interact with Smad2/3 and TGF-β receptors

A role for SARA as an adaptor protein that mediates TGF-β signaling by direct interaction with Smad2/3 and TGF-β receptors has been suggested [9], [27]. However, recent findings indicated that SARA does not associate with R-Smads and TGF-β receptors in HeLa cells [14]. To examine the interaction between endogenous mouse SARA_1/2_ and other components of the TGF-β signaling pathway, we performed immunoprecipitation analysis from TGF-β1-treated or -untreated MEFs. After immunoprecipitation with mSARA-N antibody, co-immunoprecipitates of Smad2/3 and TGF-β receptors were detected by Western blot. SARA^Δ/Δ^ MEFs were used as a negative control. It has been previously shown that 15 min of rapid TGF-β1 stimulation leads to an increase in the protein interaction between endogenous SARA and Smad2 in human mesangial cells [27]. However, we could not detect Smad2/3 or TGF-β receptors in the SARA_1/2_-immunoprecipitated complexes from TGF-β1-treated or -untreated MEFs (Figure S1A). Treatment of TGF-β1 for 5 or 15 minutes increased p-Smad2 in WT MEFs indicating that TGF-β1 stimulation in this particular experiment was really working (Figure S1B). Our data showed that no significant protein-protein interaction between endogenous mouse SARA_1/2_ and TGF-β receptors or Smad2/3 occurred in MEFs even after activation by TGF-β.

### Loss of FYVE domain of SARA does not affect the internalization of TGF-β receptors into the early endosome

**Figure 5:**
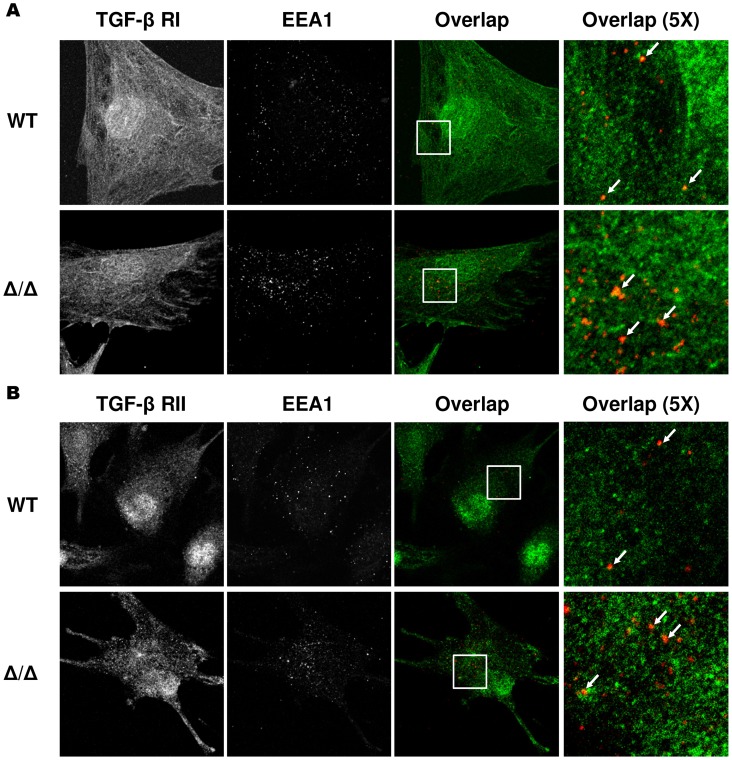
Loss of FYVE domain of SARA does not affect the internalization of TGF-β receptors into the early endosome. WT and SARA^Δ/Δ^ MEFs were incubated at 4°C for 1 hour and then treated with 4 ng/mL TGF-β1 for 30 minutes at 37°C. Cells were fixed and stained with antibodies to endogenous EEA1, TGF-β RI (A), and RII (B). The overlap between the two signals is displayed in yellow (indicated by arrows).

Internalization of TGF-β receptors into the early endosome through a clathrin-dependent pathway regulates TGF-β signaling [28]–[31]. Several reports have demonstrated that SARA facilitates the entrance of TGF-β receptors into this compartment [29], [30], which suggests that TGF-β receptor localization and colocalization with early endosome antigen 1 (EEA1), an early endosome marker, might be affected by the loss of SARA. To assess this, the localization of endogenous TGF-β receptors and EEA1 in MEFs was examined using immunofluorescence confocal microscopy. Similar to previous reports, both TGF-β RI and RII colocalized with EEA1 in WT MEFs after 30 minutes of TGF-β1 treatment (Figure 5A–B, upper panel) [29], [30], [32]. Surprisingly, the colocalization between TGF-β RI or RII and EEA1 still persisted in SARA^Δ/Δ^ MEFs, similar to WT cells (Figure 5A–B, lower panel). We were unable to detect any obvious punctate EEA1 staining pattern in non TGF-β1-stimulated MEF controls (Figure S2). These results suggest that SARA is not essential for the internalization of TGF-β receptors into EEA1-containing early endosomes.

### SARA does not participate in TGF-β-mediated cellular responses

**Figure 6:**
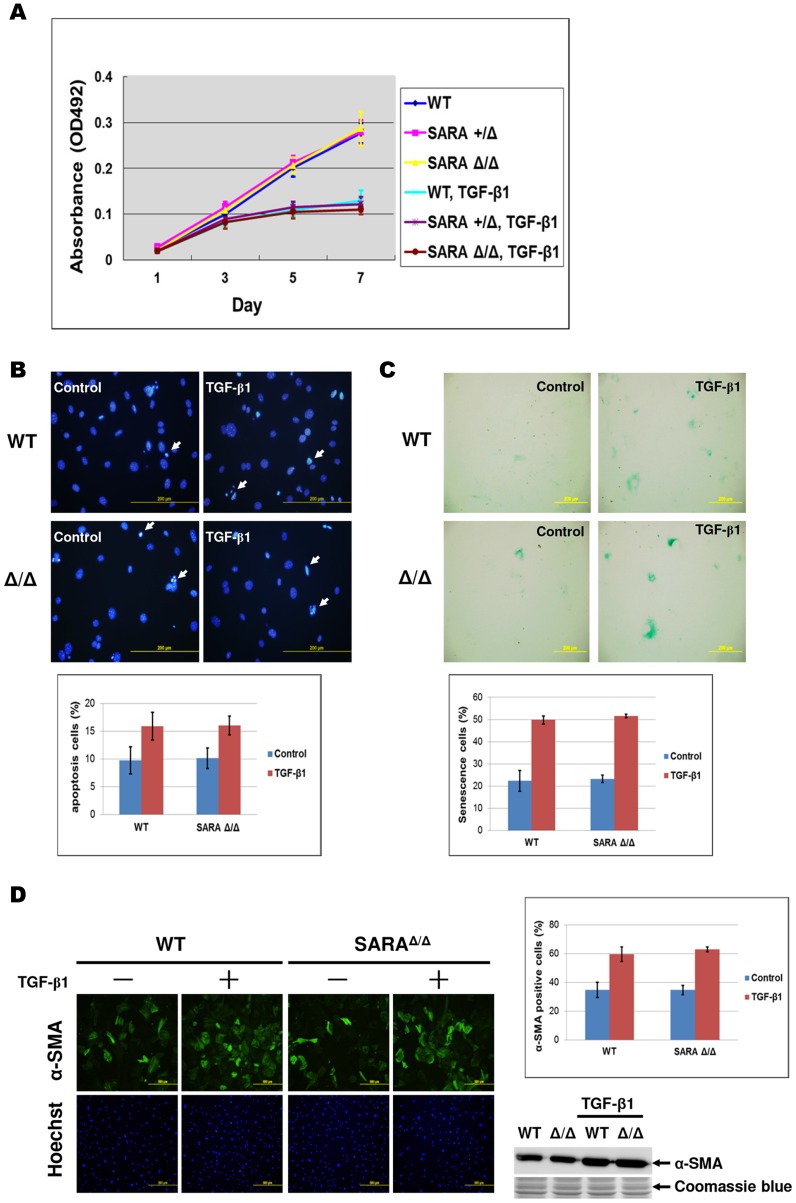
SARA does not participate in TGF-β-mediated cellular responses. (**A**) Growth curves of MEFs were determined using the MTT assay. Cells were treated with or without 4 ng/mL TGF-β1 and stained with MTT at the times indicated. The data points are the average of three independent measurements, and the standard deviation from the mean is shown. (**B**) The morphology of apoptotic cells was observed by Hoechst 33342 staining. MEFs were serum-starved for 24 hours and then treated with or without 10 ng/mL TGF-β1 for 24 hours. Apoptotic cells showed condensed chromatin and a fragmented apoptotic nucleus (indicated by arrows). The percentage of apoptotic cells was counted in ten random fields for each triplicate sample. (**C**) MEFs were treated with or without 4 ng/mL TGF-β1 for 6 days, fixed, and stained with β-gal. The percentage of senescent cells was counted in ten random fields for each triplicate sample. (**D**) MEFs were serum-starved for 24 hours and then treated with or without 10 ng/mL TGF-β1 for 3 days. Cells were subjected to immunocytochemistry and Western blot analysis using an anti-α-SMA antibody. The percentage of α-SMA positive cells was counted in ten random fields for each triplicate sample. Coomassie blue stain penicillin-streptomycinserved as the loading control.

TGF-β signaling regulates diverse cellular processes, such as cell growth inhibition, apoptosis, senescence, and the epithelial-to-mesenchymal transition (EMT) [3], [33]–[35]. Therefore, we assessed whether TGF-β-mediated cellular functions were impaired in SARA mutant MEFs. Cell proliferation was measured using the MTT assay. In the absence of TGF-β1 treatment, there was no significant difference in the cell proliferation rate between WT and SARA mutant MEFs. SARA mutant MEFs, like WT control cells, remained sensitive to the growth inhibitory effect of TGF-β1 (Figure 6A). TGF-β-mediated apoptosis was assayed by Hoechst 33342 staining. We observed no difference between SARA mutant and WT MEFs (Figure 6B). The extent of TGF-β-induced cellular senescence was determined by the senescence-associated β-galactosidase (SA-β-Gal) assay. Both WT and SARA mutant MEFs underwent senescence at similar levels after TGF-β1 treatment (Figure 6C). TGF-β is a major regulator of the EMT, and it induces smooth muscle α-actin (α-SMA) expression, a putative indicator of myofibroblast differentiation from fibroblasts [36], [37]. To examine the effect of SARA on TGF-β-induced EMT, α-SMA expression was measured in MEFs by immunocytochemistry and Western blot assays. In a previous study, knockdown of SARA led to increased α-SMA expression; it was proposed to be a key mediator of TGF-β-induced EMT [13]. However, our data showed that loss of SARA did not induce α-SMA expression in MEFs compared with WT MEFs. Moreover, TGF-β induced a similar level ofα-SMA expression in SARA mutant and WT MEFs (Figure 6D). Taken together, our data showed that the TGF-β-mediated biological functions, including growth inhibition, apoptosis, senescence, and EMT, were not significantly different in SARA mutant and WT MEFs. Thus, we conclude that SARA does not contribute to the functional TGF-β signaling, if not all, at least in primary cultured MEFs.

### SARA prevents Smurf2-induced Smad2 degradation

**Figure 7:**
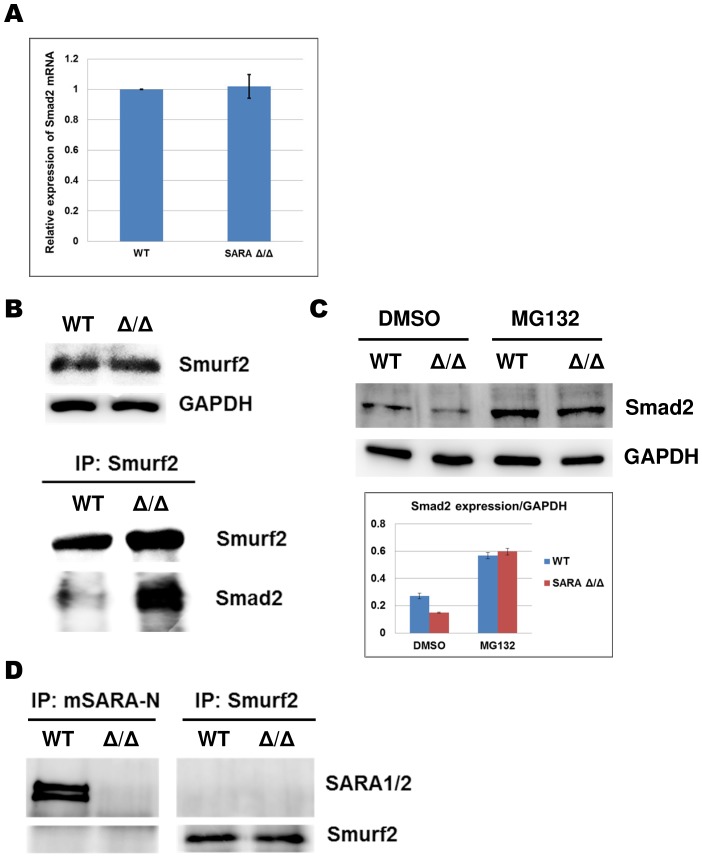
SARA prevents Smurf2-induced Smad2 degradation. (**A**) Smad2 RNA expression levels in WT and SARA mutant MEFs were quantified by Q-PCR. Data represent means of three independent experiments performed in triplicate. (**B**) Expression of Smurf2 protein in WT and SARA mutant MEFs were detected by Western blot analysis (upper panel). MEF lysates (500 µg) were immunoprecipitated (IP) with Smurf2 antibody and blotted (IB) with the indicated antibodies (lower panel). (**C**) WT and SARA mutant MEFs were treated with DMSO and MG132 (20 µM) for 8 hours prior to lysis. Expression levels of Smad2 protein were quantified by Western blot analysis. (**D**) MEF lysates (500 µg) were immunoprecipitated (IP) with Smurf2 or mSARA-N antibody and blotted (IB) with the indicated antibodies.

We next sought to characterize the mechanism of Smad2 downregulation in SARA mutant mice. Q-PCR data showed no significant changes in Smad2 mRNA expression between WT and SARA mutant MEFs (Figure 7A). It indicates that Smad2 might be regulated by SARA at the protein level rather than at the transcriptional level. Smurf2 is an ubiquitin E3 ligase that targets Smad2 for proteasome-dependent degradation [38], [39]. A previous in vitro study suggested that the downregulated SARA expression enhanced Smad2 and Smurf2 protein-protein interaction, thereby facilitating Smad2 protein degradation [13]. To test whether Smad2 protein could be mediated by the same pathway *in vivo*, we analyzed the interaction between Smad2 and Smurf2 protein using SARA mutant MEFs. Despite equal levels of Smurf2 protein expression between WT and SRAR-KO MEFs (Figure 7B, upper panel), Smad2 and Smurf2 protein-protein interaction is dramatically enhanced in SARA mutant MEFs (Figure 7B, lower panel). Furthermore, degradation of the Smad2 protein in SARA mutant MEFs was inhibited by the proteasome inhibitor MG132, which is suggested to block proteasomal degradation (Figure 7C). These data suggest that the reduced expression of Smad2 in SARA mutant MEFs is dependent on Smurf2-mediated proteasomal degradation pathway. However, SARA was unable to interact with Smad2 directly in SARA mutant MEFs (Figure S1). Therefore, we sought to determine if there are protein-protein interaction between SARA and Smurf2. The immunoprecipitation data showed that no interaction could be detected between SARA and Smurf2, no matter which protein is immunoprecipitated in MEFs (Figure 7D). These results suggest that SARA possibly regulates Smad2 protein degradation through an indirect mechanism that does not involve binding to Smad2 or Smurf2 proteins.

### Loss of SARA promotes skin tumor formation and malignant progression

**Figure 8:**
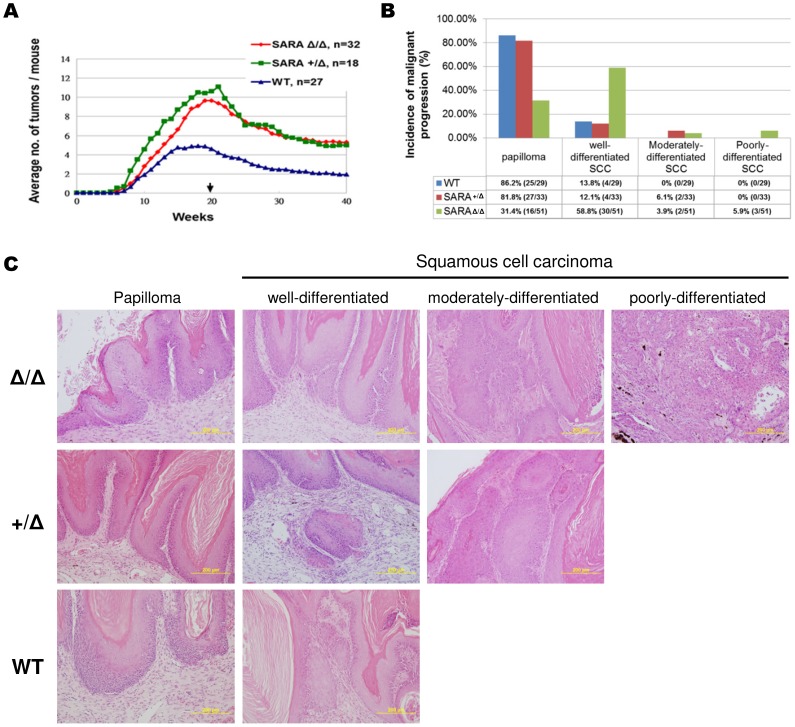
Loss of SARA promotes skin tumor formation and malignant progression. (**A**) Average number of tumors in WT and SARA mutant mice at different time points. Arrow indicates TPA withdrawal. Twenty weeks after the beginning of promotion, a significant difference in the number of papillomas per mouse between the WT and SARA mutant mice were evident (*P*<0.05). (**B**) Incidence of malignant progression in skin tumors generated 40 weeks after the beginning of promotion. SARA^Δ/Δ^ mice showed a highly significant increase in the percentage of SCCs compared with SARA^+/Δ^ and WT controls. Moderately- and poorly-differentiated SCCs were not found in WT mice. (**C**) Histological analysis of skin tumors. Papilloma (note a proliferation of hyperkeratotic stratified squamous epithelium); well-differentiated SCCs (note tumor cells destroy the basement membrane and invade the dermis); moderately-differentiated SCCs (note cells are markedly irregular in shape and size, distinct nuclear pleomorphism and mitotic activity); and poorly-differentiated SCCs (note immature cells predominate, with numerous atypical mitosis, and minimal keratinization) are shown.

In SARA mutant mice, a significant decrease in Smad2 protein was detected in MEFs and skin tissues (Figure 4). Keratinocyte-specific Smad2-KO mice display accelerated skin tumor formation and progression [21]. To test whether SARA played a causal role in skin carcinogenesis, SARA mutant and WT mice were challenged using a two-stage chemically induced carcinogenesis protocol with DMBA as the initiator, followed by twice weekly treatments with the tumor promoter TPA. The papilloma number was 2-fold higher in SARA^+/Δ^ and SARA^Δ/Δ^ mice than in WT mice (Figure 8A), indicating that SARA ablation promoted tumor formation. Forty weeks after the beginning of promotion, 86.2% of WT tumors were benign papillomas and 13.8% of tumors had progressed to well-differentiated squamous cell carcinoma (SCC) (Figure 8B and C). The incidence of malignant tumors in SARA^+/Δ^ mice was similar or slightly increased: 12.1% of SARA^+/Δ^ tumors exhibited well-differentiated SCCs and 6.1% showed moderately-differentiated SCCs (Figure 8B and C). However, it is noteworthy that while loss of both SARA alleles, most of the benign papillomas were converted into malignant forms: 58.8%, 3.9%, and 5.9% of tumors in SARA^Δ/Δ^ mice showed well-differentiated, moderately-differentiated, and poorly-differentiated SCCs, respectively (Figure 8B and C). Taken together, our results indicate that loss of SARA not only promotes skin tumor formation, but also stimulates malignant transformation.

## Discussion

The expression and biochemical function of human SARA has been described using some *in vitro* cell systems. SARA contains three important domains (N-terminal FYVE, Smad binding domain, and C-terminal receptor binding domain) and serves as a scaffold protein for assembling the complex of TGF-β receptors and Smad2/3 at cell membrane regions enriched in PtdIns3P [9], [10]. Subcellular internalization of the TGF-β receptors/SARA/Smads complex into early endosomes facilitates Smad2/3 phosphorylation and enhances TGF-β signaling [12], [27], [28]. Mutations of SARA resulted in mislocalization of Smad2 and loss of TGF-β signaling [9], [10]. Thus, SARA is considered essential for TGF-β signaling transduction and activation. However, this model has been challenged by other reports. First, SARA seems to preferentially interact with Smad2 rather than with Smad3 [27], and it participates in TGF-β/Smad2 signaling due to maintenance of Smad2 expression but not Smad3 [13]. Second, another report showed that SARA is not essential for TGF-β-mediatedSmad2 [16] or Smad3 signaling [15]. Third, subcellular internalization of the activated receptor-bound complex is not required for TGF-β signaling [16]. Fourth, SARA does not interact with TGF-β receptors or Smad2/3, and it is dispensable for TGF-β signaling in some cell lines [14]. Therefore, the specific role of SARA in TGF-β signaling is controversial. Notably, all of these data came from *in vitro* cell culture assays that primarily use overexpression systems, which may not accurately reflect the *in vivo* functions of SARA. To investigate the biochemical and biological significance of SARA in organisms, we used a loss-of-function approach in a mouse model to clarify these issues. This is the first study to describe the biological and biochemical roles of SARA *in vivo*.

We found that mouse SARA exists in two isoforms, a full-length transcript (SARA_1_) and a shorter transcript lacking the Smad binding domain (SARA_2_); the Ensembl-predicted isoform lacking the FYVE domain (SARA_3_) was not found. By analyzing the expression pattern of SARA in mice, we demonstrated that SARA_1_ and SARA_2_ are ubiquitously expressed at different levels in mouse tissues from early embryonic stages through adulthood. In addition, the distinct protein expression ratios between SARA_1_ and SARA_2_ in different organs (Figure 1B) suggest that different SARA isoforms may play distinct roles. The difference between these two proteins is the presence of the Smad binding domain in SARA_1_, which facilitates binding of Smad2/3 to the receptors. Thus, the unexpected presence of endogenous SARA_2_ in organisms implies that there may be a different role for SARA independent of an interaction with Smad proteins.

Since previous studies have suggested that SARA plays a critical role in TGF-β signaling in cell lines, we speculated that SARA deficiency would cause defects in mouse development. However, no significant abnormalities were detected in SARA mutant mice during development. Nevertheless, this finding may be due to compensation by other FYVE family members, such as Hrs (hepatic growth factor-regulated tyrosine kinase substrate). Hrs plays an essential role in mouse early development [40], [41] and contributes to TGF-β signaling through cooperation with SARA [40].

Furthermore, our SARA mutant mice produced extra SARA truncated protein products (SARA_3_ and SARA_4_) from the targeted allele. Therefore, we could not exclude the possibility that these mutant proteins contain enough activity to maintain embryonic development and growth. Previous studies have shown that SARA protein lacking FYVE domain abrogates SARA's ability to initiate signal transduction as a dominant-negative inhibitor [9], [12], [31]. Despite the lack of significant developmental abnormalities, SARA mutant mice still harbored some defects in SARA-mediated TGF-β signal transduction. Further analysis confirmed that TGF-β-mediated Smad2 protein phosphorylation and nuclear translocation were downregulated in SARA mutant mice. Of note, the downregulation in TGF-β signaling was caused by decreased protein levels of Smad2, not by an impaired ability to transduce TGF-β signals. This phenomenon was not observed with Smad3.

Because signal transduction ability of TGF-β pathway is intact in SARA mutant mice, we hypothesized that SARA may not directly participate in the transduction of TGF-β signaling. Immunoprecipitation analysis revealed that neither TGF-β receptors nor Smad2/3 interact with SARA_1_ or SARA_2_ in the absence or presence of TGF-β stimulation. This result is consistent with the findings of a previous study [14]. The results of colocalization analyses of TGF-β receptors with EEA1 further confirmed that neither SARA_1_ nor SARA_2_ is essential for TGF-β receptor internalization into early endosomal compartments. Cytoplasmic promyelocytic leukemia (cPML) is an essential regulator of TGF-β signaling; it interacts with the TGF-β receptor/SARA/Smad complex and facilitates internalization of this complex into the early endosomes. Loss of cPML resulted in mislocalization of SARA and TGF-β receptors with EEA1 and further attenuated TGF-β signaling transduction [32]. In contrast, SARA deficiency did not cause mislocalization of TGF-β receptors with EEA1. These results indicate that cPML is a key component in the regulation of TGF-β receptor internalization, whereas SARA probably is not. Based on these results, we conclude that SARA is not required for the activation process of TGF-β signal transduction; rather, it modifies TGF-β signaling by regulating the expression of Smad2 protein.

Since loss of SARA decreased the protein expression of Smad2, we examined whether TGF-β-mediated biological functions were impaired in SARA mutant mice. We observed no detectable effects of SARA on TGF-β-mediated growth inhibition, apoptosis, senescence, or the EMT using primary cultured MEFs as an *in vitro* model. Similar results were obtained in SARA mutant mice; no significant TGF-β-related phenotypes have been observed in SARA mutant mice to date. These data indicate that the loss of SARA, despite decreasing Smad2 expression, is not sufficient to exert a negative influence on MEFs in response to TGF-βsignaling. Loss of SARA expression results in a concomitant decrease in Smad2 expression in some epithelial cell lines, and it may enhance marker expression (α-SMA) consistent with EMT [13]. However, we did not observe enhanced α-SMA expression in SARA mutant MEFs. The discrepancy may be due to the use of different cell types or differences between primary cultured cells and an immortal cell line.

Following investigation showed that Smad2 was targeted for degradation via the Smurf2-mediated ubiquitin-proteasome system in SARA mutant MEFs. This result is consistent with the findings of a previous in vitro study [13]. Smurf2 is a HECT class ubiquitin E3 ligase that induces the ubiquitination and degradation of TGF-β-induced p-Smad2 [38], [39]. It has been found that ubiquitination and degradation of nuclear Smad2 is caused by Smad2 accumulates in the nucleus and is independent from the phosphorylation of Smad2 [42]. In SARA mutant mice, we detected a significant enhanced protein-protein interaction between Smad2 and Smurf2 but not the protein amount of Smurf, suggesting that an abnormal accumulation of Smad2 in the nucleus. It has been showed that Smad2 would be maintained in cytoplasm by interacting with SARA and can be released to nucleus by the phosphorylation through TGF-β signal transduction [43], [44]. Interestingly, in our study, we found that loss of SARA does not affect the ability of canonical TGF-β signaling pathway to induce phosphorylation of Smad2. In addition, the interaction between SARA and Smad2 was not detected in our MEF cells. Moreover, only FYVE-domain of SARA was deleted in SARA mutant mice should only affected the membrane-binding affinity of SARA. How this membrane-binding-deficient mutant SARA could unmask the nuclear import function of Smad2 in our *in vivo* SARA mutant mice is still unclear. The detailed molecular mechanisms require further investigation.

Dysfunction of TGF-β signaling is associated with a variety of human pathologies, such as fibrosis and cancer. Studies carried out with a two-stage carcinogenesis model and some *in vitro* cell culture systems have provided abundant evidence that TGF-β has dual roles in cancer. During early tumor progression, TGF-β first acts as a tumor suppressor and later as a tumor promoter at tumor malignant conversion. It acts directly on tumor cells to enhance the EMT [3], [45], [46]. In contrast, loss of Smad2 in keratinocytes can accelerate skin tumor formation in the early stages and increase malignant conversion in the later stages [21]. Unlike the dual effects of TGF-β, Smad2 has inhibitory effects on both early tumor growth and later malignant conversion. A previous study suggested that prolonged treatment with TGF-β reduces SARA expression, further decreases Smad2 protein, and enhances the EMT phenotype [13]. Based on its critical role in TGF-β signaling transduction, SARA has been suspected as a key factor in tumorigenesis. Here, we have provided the first evidence that loss of SARA causes tumor growth in early stages, and promotes the malignant conversion in later stages in a skin cancer animal model. SARA mutant mice had decreased levels of Smad2 protein in skin tissue and exhibited a similar tumorigenic phenotype as keratinocyte-specific Smad2-KO mice, indicating that SARA may exert its tumor suppressive effects in part through modulation of Smad2 protein levels. Notably, the loss of one Smad2 allele is sufficient to promote tumor formation and malignant progression [21]. In addition, loss of one SARA allele in mice, which express more Smad2 protein than in Smad2^+/−^ mice, only induced tumor formation but not highly malignant progression. Our results suggest that malignant conversion requires a significantly lower level of Smad2 than tumor formation. Thus, we conclude that SARA may play a role in the maintenance of the Smad2 checkpoint activity that is required to block not only tumor formation but also malignant conversion at different threshold concentrations.

TGF-β signaling generally is a master immunosuppressive regulator of the immune response. It inhibits both B and T lymphocyte proliferation and differentiation, and alters the functions of all classes of mature leukocytes [47]–[49]. In addition to the alteration of TGF-β signaling pathway in skin cancer cells, the innate and adaptive immune systems also play a critical role in tumor growth and progression [50]. Although our SARA mutant mice exhibit symptoms similar to keratinocyte-specific Smad2-KO mice, we could not rule out whether SARA mutant mice have some defects in their immune system which may contribute to promote tumorigenesis. To figure out this question, further experiments must be carried.

The importance of TGF-β-induced EMT in the malignant conversion of cancer has been demonstrated. These conversion changes are reversible upon removal of TGF-β. Thus, many different cancer therapy approaches involving the inhibition of TGF-β-induced EMT have been considered. Although tumor invasion and metastasis is reduced by inhibiting TGF-β signaling, the loss of TGF-β-mediated growth inhibition on other normal cells often increases the risk of tumor formation. This dual role of TGF-β could pose a challenge when targeting the TGF-β signaling system for cancer treatment. Recent studies showed that Smad2 has a tumor suppressive effect on both tumor growth and malignant conversion, thus making it a promising candidate for cancer therapy. However, overexpression of Smad2 upregulated TGF-β signaling [51], which may affect the immune system and normal cell functions. Interestingly, SARA also has a tumor suppressive effect on both tumor growth and malignant conversion. Unlike Smad2, however, overexpression of SARA in cultured cells did not alter TGF-β signaling [9], [40]. Thus, it is worthy to know that SARA could be an attractive novel target for cancer therapy. However, it is still unclear how SARA deficiency promotes skin carcinogenesis and how TGF-β mediates the loss of SARA expression. The answers to these questions may give us more insight into the possibilities of this novel therapeutic target and strategies for cancer treatment.

## Supporting Information

Figure S1 **SARA does not interact with Smad2/3 and TGF-β receptors.** (**A**) MEFs were treated with or without 4 ng/mL TGF-β1 for 5 or 15 minutes. MEF lysates (500 µg) were immunoprecipitated (IP) with anti-mouse SARA-N antibody and blotted (IB) with the indicated antibodies. SARA^Δ/Δ^ MEF served as the negative control. WT MEF lysate (100 µg) served as the input control. (B) WT MEFs were treated with or without 4 ng/mL TGF-β1 for 5 or 15 minutes. Phosphorylation of Smad2 (p-Smad2) was detected by Western blot. GAPDH served as the input control.
(TIF)

Figure S2 **Punctate EEA1 staining pattern is not exhibited in non TGF-β1-stimulated MEF controls.** WT and SARAΔ/Δ MEFs were incubated at 4°C for 1 hour and then 37°C for 30 minutes. Cells were fixed and stained with antibodies to endogenous EEA1, TGF-β RI, and RII.
(TIF)

Table S1 **Offspring from SASA^+/Δ^ intercrosses are born at Mendelian frequencies.**
(TIF)

Text S1 **cDNA sequences of SARA variant transcripts.**
(DOCX)
