# Supplementary figures and images for: The FYVE Domain of Smad Anchor for Receptor Activation (SARA) Is Required to Prevent Skin Carcinogenesis, but Not in Mouse Development
Source: PLoS One. 2014 Aug 29;9(8):e105299. doi: 10.1371/journal.pone.0105299 (PMC4149420; doi:10.1371/journal.pone.0105299)

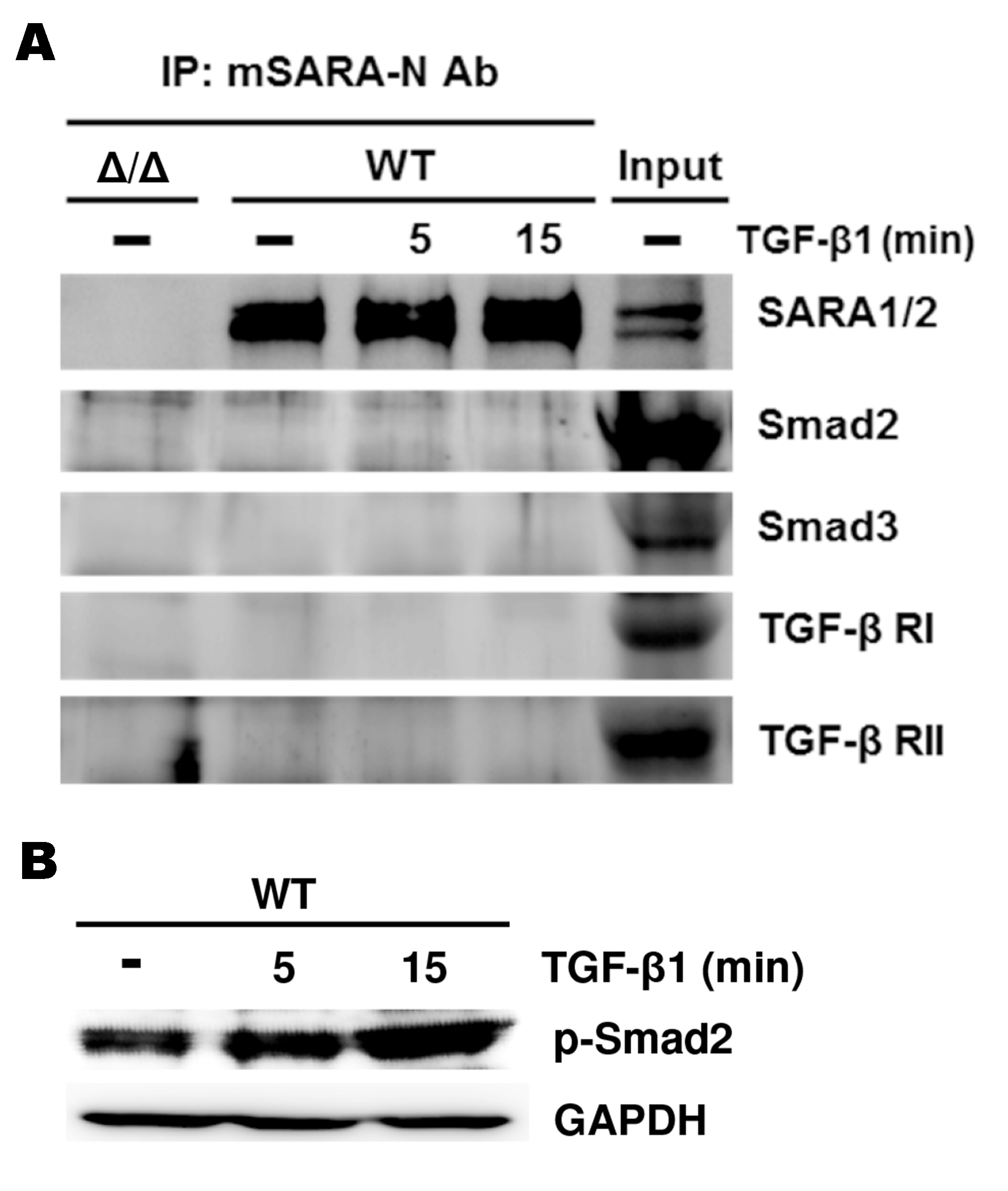

Supplement: Figure S1 — SARA does not interact with Smad2/3 and TGF-β receptors. (A) MEFs were treated with or without 4 ng/mL TGF-β1 for 5 or 15 minutes. MEF lysates (500 µg) were immunoprecipitated (IP) with anti-mouse SARA-N antibody and blotted (IB) with the indicated antibodies. SARAΔ/Δ MEF served as the negative control. WT MEF lysate (100 µg) served as the input control. (B) WT MEFs were treated with or without 4 ng/mL TGF-β1 for 5 or 15 minutes. Phosphorylation of Smad2 (p-Smad2) was detected by Western blot. GAPDH served as the input control. (TIF) [file pone.0105299.s001.tif]

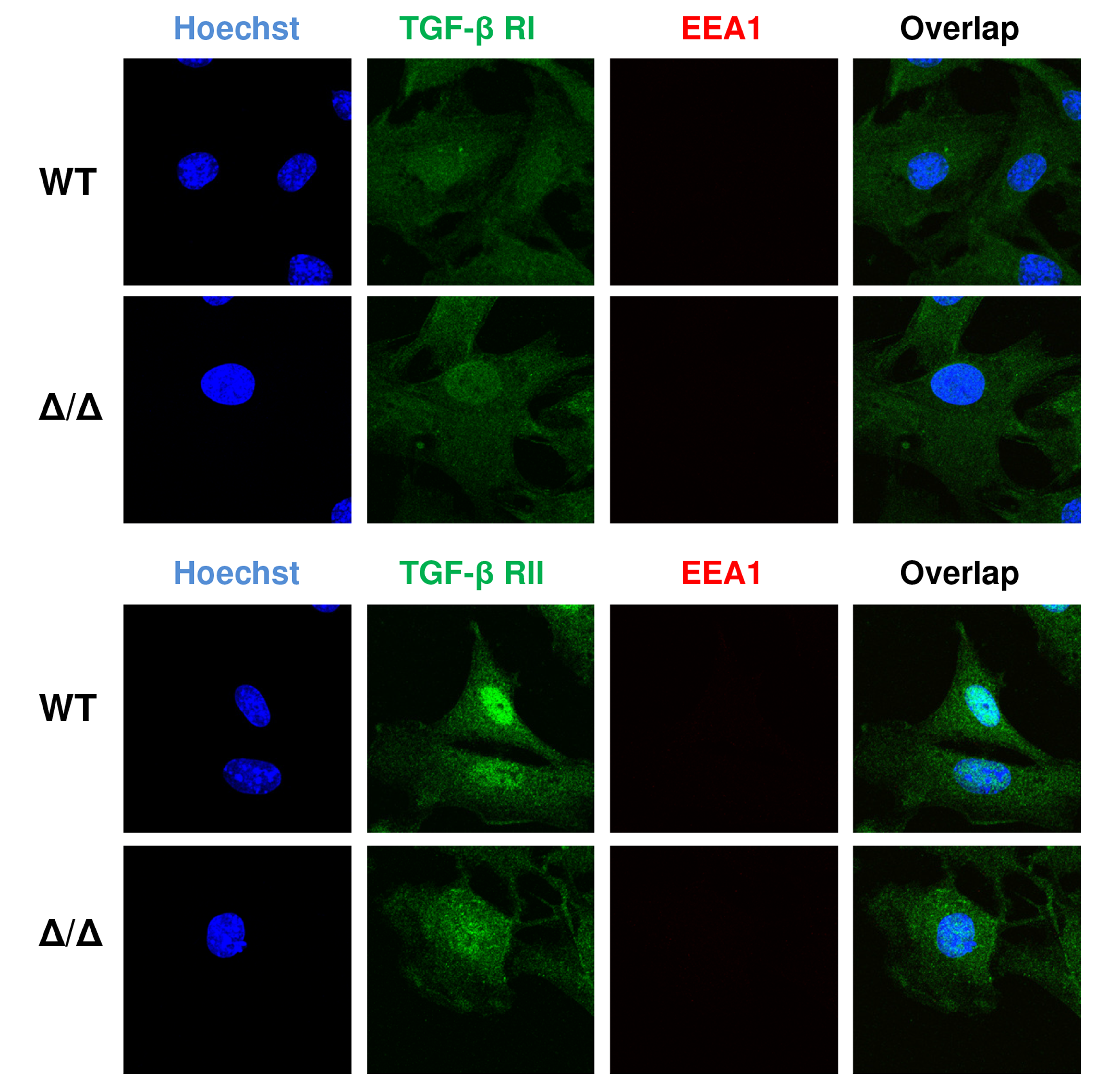

Supplement: Figure S2 — Punctate EEA1 staining pattern is not exhibited in non TGF-β1-stimulated MEF controls. WT and SARAΔ/Δ MEFs were incubated at 4°C for 1 hour and then 37°C for 30 minutes. Cells were fixed and stained with antibodies to endogenous EEA1, TGF-β RI, and RII. (TIF) [file pone.0105299.s002.tif]

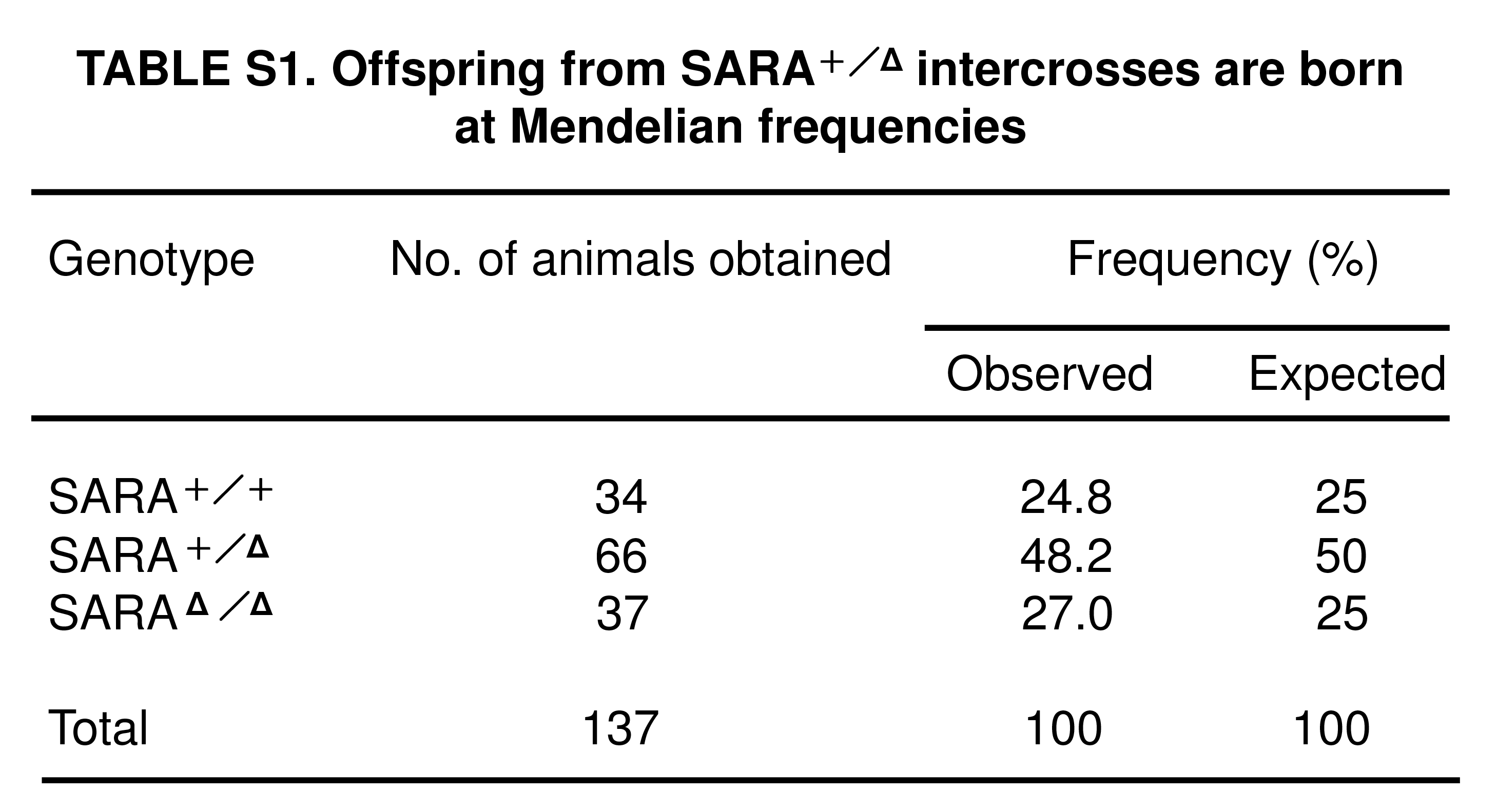

Supplement: Table S1 — Offspring from SASA+/Δ intercrosses are born at Mendelian frequencies. (TIF) [file pone.0105299.s003.tif]
