# Supplementary material for: The FYVE Domain of Smad Anchor for Receptor Activation (SARA) Is Required to Prevent Skin Carcinogenesis, but Not in Mouse Development
Source: PLoS One. 2014 Aug 29;9(8):e105299. doi: 10.1371/journal.pone.0105299 (PMC4149420; doi:10.1371/journal.pone.0105299)
Supplement: Text S1 — cDNA sequences of SARA variant transcripts. (DOCX) [file pone.0105299.s004.docx]

SARA_1_ (1397 a.a.) (Protein ID: ENSMUSP00000102268);

MENYFQAEAYNLDKVLDEFEQNEDETVSPTLLDTKWNKILDPSSHPLSFNPALASVNEPTVSETGPQLKVFSLAGSAPLTKEDKDPCANGQDCSLNPETDTMWIDENAVTEDQLIKRNYNQDDQFSAVEVGEEKCGSLTCLPDEKNVLVVAVMHNCDKRTLQSDLQDCNNYNSQSLMNSFSCSLDNETRQTDQFSFSMNGSTEKGINSEKQMDALNKPKPERDSVNHLCAASSNSATSISSPSQLKDGENVGRDPSTSTVTSLAVNSSQGMDGGPAIKQQGNYMPDEDLSGMNSSSRTDLGISNSFSHSSGELLIKTEPAEERTAEDSLPSDLSLNLKPDTPALSGRDNCEQSSDCLGSSETRADEDEGNDSQMTNWKLTKLNEMSDSQVNEENQMALQSNQPEDTNSGGECVGMADSDLDFKGTCMNESEGYDFSTVNDAPAANSLSNSCDSYGMQSPIVSFVPKTLPSKEDSVTEEKEIEESKSECYSNIYEQRGNENAEGSGLLLNSTGNVMKKNYLHNFCSQIPSVLGQSSPKIANLQSISVPFGGARPKQPSNLKLQIPKPLSDHLQNDLPANNGNNSKNKNDVLGKAKLGENSAVNACSATLGNISVTDTNGEHLETYEAEISSRPCLALAPDSPDNDLRASQFGISARKPFTTLGEVAPVWVPDSQAPNCMKCEARFTFTKRRHHCRACGKVFCASCCSLKCKLLYMDRKEARVCVICHSVLMNAQAWENMMSASSQSPNPNNPAEYCSTIPPLQQAQASGALSSPPPTVMVPVGVLKHPGTEVPQPREQRRVWFADGILPNGEVADAAKLTMNGTSSAGTLAVSHDPVKPVATSPLPTEADTSLFSGSITQVGSPVGSAMNLIPEDGLPPILISTGVKGDYAVEEKPSQISVMQQLEDGGPDPLVFVLNANLLSMVKIVNYVNRKCWCFTTKGMHAVGQSEIVILLQCLPDEKCLPKDIFNHFVQLYRDALAGNVVGSLGHSFFSQSFLGSKEHGGFLYVTSTYQSLQDLVLPTPPYLFGILIQKWETPWAKVFPIRLLLRLGAEYRLYPCPLFSVRFRKPLFGETGHTIMNLLADFRNYQYTLPVVQGLVVDMEVRKTSIKIPSNRYNEMMKAMNKSNEHVLAGGACFNEKADSHLVCVQNDDGNYQTQAISIHNQPRKVTGASFFVFSGALKSSSGYLAKSSIVEDGVMVQITAENMDSLRQALREMKDFTITCGKADAEDPQEQIHIQWVDDDKTVNKGVVSPIDGKSMESITNVKIFHGSEYKANGKVIRWTEVFFLENDDHHNCLSDPADHSRLTEHVAKAFCLALCPHLKLLKEDGMTKLGLRVTLDSDQVGYQAGSNGQPLPSQYMNDLDSALVPVIHGGACQLSEGPVVMELIFYILENIA

SARA_2_ (1338 a.a.) (Protein ID: ENSMUSP00000102269)

MENYFQAEAYNLDKVLDEFEQNEDETVSPTLLDTKWNKILDPSSHPLSFNPALASVNEPTVSETGPQLKVFSLAGSAPLTKEDKDPCANGQDCSLNPETDTMWIDENAVTEDQLIKRNYNQDDQFSAVEVGEEKCGSLTCLPDEKNVLVVAVMHNCDKRTLQSDLQDCNNYNSQSLMNSFSCSLDNETRQTDQFSFSMNGSTEKGINSEKQMDALNKPKPERDSVNHLCAASSNSATSISSPSQLKDGENVGRDPSTSTVTSLAVNSSQGMDGGPAIKQQGNYMPDEDLSGMNSSSRTDLGISNSFSHSSGELLIKTEPAEERTAEDSLPSDLSLNLKPDTPALSGRDNCEQSSDCLGSSETRADEDEGNDSQMTNWKLTKLNEMSDSQVNEENQMALQSNQPEDTNSGGECVGMADSDLDFKGTCMNESEGYDFSTVNDAPAANSLSNSCDSYGMQSPIVSFVPKTLPSKEDSVTEEKEIEESKSECYSNIYEQRGNENAEGSGLLLNSTGNVMKKNYLHNFCSQIPSVLGQSSPKIANLQSISVPFGGARPKQPSNLKLQIPKPLSDHLQNDLPANNGNNSKNKNDVLGKAKLGENSAVNACSATLGNISVTDTNGEHLETYEAEISSRPCLALAPDSPDNDLRASQFGISARKPFTTLGEVAPVWVPDSQAPNCMKCEARFTFTKRRHHCRACGKVFCASCCSLKCKLLYMDRKEARVCVICHSVLMNVPQPREQRRVWFADGILPNGEVADAAKLTMNGTSSAGTLAVSHDPVKPVATSPLPTEADTSLFSGSITQVGSPVGSAMNLIPEDGLPPILISTGVKGDYAVEEKPSQISVMQQLEDGGPDPLVFVLNANLLSMVKIVNYVNRKCWCFTTKGMHAVGQSEIVILLQCLPDEKCLPKDIFNHFVQLYRDALAGNVVGSLGHSFFSQSFLGSKEHGGFLYVTSTYQSLQDLVLPTPPYLFGILIQKWETPWAKVFPIRLLLRLGAEYRLYPCPLFSVRFRKPLFGETGHTIMNLLADFRNYQYTLPVVQGLVVDMEVRKTSIKIPSNRYNEMMKAMNKSNEHVLAGGACFNEKADSHLVCVQNDDGNYQTQAISIHNQPRKVTGASFFVFSGALKSSSGYLAKSSIVEDGVMVQITAENMDSLRQALREMKDFTITCGKADAEDPQEQIHIQWVDDDKTVNKGVVSPIDGKSMESITNVKIFHGSEYKANGKVIRWTEVFFLENDDHHNCLSDPADHSRLTEHVAKAFCLALCPHLKLLKEDGMTKLGLRVTLDSDQVGYQAGSNGQPLPSQYMNDLDSALVPVIHGGACQLSEGPVVMELIFYILENIA

SARA_3_ (706 a.a.) (Protein ID: ENSMUSP00000039852)

MNLNKMKVFCASCCSLKCKLLYMDRKEARVCVICHSVLMNAQAWENMMSASSQSPNPNNPAEYCSTIPPLQQAQASGALSSPPPTVMVPVGVLKHPGTEVPQPREQRRVWFADGILPNGEVADAAKLTMNGTSSAGTLAVSHDPVKPVATSPLPTEADTSLFSGSITQVGSPVGSAMNLIPEDGLPPILISTGVKGDYAVEEKPSQISVMQQLEDGGPDPLVFVLNANLLSMVKIVNYVNRKCWCFTTKGMHAVGQSEIVILLQCLPDEKCLPKDIFNHFVQLYRDALAGNVVGSLGHSFFSQSFLGSKEHGGFLYVTSTYQSLQDLVLPTPPYLFGILIQKWETPWAKVFPIRLLLRLGAEYRLYPCPLFSVRFRKPLFGETGHTIMNLLADFRNYQYTLPVVQGLVVDMEVRKTSIKIPSNRYNEMMKAMNKSNEHVLAGGACFNEKADSHLVCVQNDDGNYQTQAISIHNQPRKVTGASFFVFSGALKSSSGYLAKSSIVEDGVMVQITAENMDSLRQALREMKDFTITCGKADAEDPQEQIHIQWVDDDKTVNKGVVSPIDGKSMESITNVKIFHGSEYKANGKVIRWTEVFFLENDDHHNCLSDPADHSRLTEHVAKAFCLALCPHLKLLKEDGMTKLGLRVTLDSDQVGYQAGSNGQPLPSQYMNDLDSALVPVIHGGACQLSEGPVVMELIFYILENIA

SARA_4_ (647 a.a.)

MNLNKMKVFCASCCSLKCKLLYMDRKEARVCVICHSVLMNVPQPREQRRVWFADGILPNGEVADAAKLTMNGTSSAGTLAVSHDPVKPVATSPLPTEADTSLFSGSITQVGSPVGSAMNLIPEDGLPPILISTGVKGDYAVEEKPSQISVMQQLEDGGPDPLVFVLNANLLSMVKIVNYVNRKCWCFTTKGMHAVGQSEIVILLQCLPDEKCLPKDIFNHFVQLYRDALAGNVVGSLGHSFFSQSFLGSKEHGGFLYVTSTYQSLQDLVLPTPPYLFGILIQKWETPWAKVFPIRLLLRLGAEYRLYPCPLFSVRFRKPLFGETGHTIMNLLADFRNYQYTLPVVQGLVVDMEVRKTSIKIPSNRYNEMMKAMNKSNEHVLAGGACFNEKADSHLVCVQNDDGNYQTQAISIHNQPRKVTGASFFVFSGALKSSSGYLAKSSIVEDGVMVQITAENMDSLRQALREMKDFTITCGKADAEDPQEQIHIQWVDDDKTVNKGVVSPIDGKSMESITNVKIFHGSEYKANGKVIRWTEVFFLENDDHHNCLSDPADHSRLTEHVAKAFCLALCPHLKLLKEDGMTKLGLRVTLDSDQVGYQAGSNGQPLPSQYMNDLDSALVPVIHGGACQLSEGPVVMELIFYILENIA

**Purple highlighting indicates exon 1.**

**Blue highlighting indicates exon2.**

**Green highlighting indicates exon3.**

**Pink highlighting indicates exon4.**

**Red highlighting indicates amino acids encoded across a splice junction.**

**Solid underline indicates the FYVE domain.**

**Dashed underline indicates the Smad-binding domain.**
